# T2AR: trust-aware ad-hoc routing protocol for MANET

**DOI:** 10.1186/s40064-016-2667-6

**Published:** 2016-07-07

**Authors:** Gayathri Dhananjayan, Janakiraman Subbiah

**Affiliations:** Department of Computer Science, Bharathiar University, Coimbatore, Tamil Nadu 641046 India; Department of Banking Technology, Pondicherry University, Pondicherry, India

**Keywords:** Mobile ad-hoc network (MANET), Ad hoc on-demand distance vector (AODV), Trust management, Neighbor estimation algorithm

## Abstract

Secure data transfer against the malicious attacks is an important issue in an infrastructure-less independent network called mobile ad-hoc network (MANET). Trust assurance between MANET nodes is the key parameter in the high-security provision under dynamic topology variations and open wireless constraints. But, the malicious behavior of nodes reduces the trust level of the nodes that leads to an insecure data delivery. The increase in malicious attacks causes the excessive energy consumption that leads to a reduction of network lifetime. The lack of positional information update of the nodes in ad-hoc on-demand vector (AODV) protocol during the connection establishment offers less trust level between the nodes. Hence, the trust rate computation using energy and mobility models and its update are the essential tasks for secure data delivery. This paper proposes a trust-aware ad-hoc routing (T2AR) protocol to improve the trust level between the nodes in MANET. The proposed method modifies the traditional AODV routing protocol with the constraints of trust rate, energy, mobility based malicious behavior prediction. The packet sequence ID matching from the log reports of neighbor nodes determine the trust rate that avoids the malicious report generation. Besides, the direct and indirect trust observation schemes utilization increases the trust level. Besides, the received signal strength indicator utilization determines the trusted node is within the communication range or not. The comparative analysis between the proposed T2AR with the existing methods such as TRUNCMAN, RBT, GR, FBR and DICOTIDS regarding the average end-to-end delay, throughput, false positives, packet delivery ratio shows the effectiveness of T2AR in the secure MANET environment design.

## Background

Mobile ad hoc network (MANET), a dynamic configured and infrastructure less architecture that contains operating nodes responsible for data transfer. The absence of centralized controller to determine the routing path initiates the cooperation mechanism among the nodes for data packets transfer. Generally, the nodes in the MANET are responsible for packet transfer and their join or leave the network without any constraints leads to an unpredictable MANET structure. Moreover, the absence of centralized administration in MANET requires nodes cooperation with the assumption of genuine and trustworthiness. Practically, these assumptions are violated due to the user misbehavior and the denial of service (DoS) attacks generation. Besides, the arrival of malicious attacks degrade the network lifetime and disrupt the data delivery that leads to an evolution of security provision schemes in the research studies. The lack of prior information about the other nodes in cooperation mechanism leads to a sharing of resources to both trusted and non-trusted nodes. Hence, there is a need of formalization of trustworthiness to assure the resource sharing between the trusted nodes only. The dynamic and unpredictable nature of MANET causes the vulnerability of several attacks that lead to less security. Trust is an important aspect of MANET to create the secure MANET environment. The evolution of trust management protocols to provide/enhance the trust level creates a new era in the research studies.

The security enhancement employs the trust management protocols which stimulate the cooperation behavior in two phases namely, prevention and detection based approaches. Prevention-based approaches require the centralized control that is not suitable for distributed environment. The evolution of detection approaches based on trust management protocols do not consider the malicious behavior observed from direct and indirect schemes. The trust updating and reputation protocols implementation are the active research area to suppress the effect of malicious nodes. The major factors limit the operation of nodes are power, computing ability, and battery and such affected nodes refer selfish nodes. Hence, the resource preservation is a pre-requisite to handle the selfish node operation. The mobility and relocation of selfish nodes in MANET have the great impact on malicious node avoidance. The intruder acquires the information regarding the dynamic changes due to relocation process in the routing path to provide the effective data delivery. Hence, the trust management requires an immediate attention in diverse and crucial MANET applications. But, the introduction of malicious behavior during the communication disrupt the performance of protocol performance. The provision of false information by the nodes and the opinion-based trust estimation disrupt the data delivery adversely in traditional direct and indirect observation schemes.

To overcome these problems, an energy model-based trust evaluation scheme is presented in this paper. The neighbor log collection and route maintenance based on log reports constitute the energy models with stable connections. The traditional ad-hoc on demand vector (AODV) routing protocol performs the sequential update for neighbor log collection. But, the connection establishment during the movement of nodes is not an effective due to the lack of positional update. Hence, an extension trust-aware ad-hoc routing protocol (T2AR) is proposed in this paper. The novelty lies in T2AR is that the use of direct and indirect trust observation schemes on neighbor log results and trust assurance via sequence ID matching.

The technical contributions of this paper are listed as follows:

- Neighbor sensing and route maintenance are based on the log collection from the nodes that provide the necessary trust rate value. Besides, the trust value is periodically updated through the locational information to enhance the security level of nodes present in MANET.
- The utilization of direct/indirect observation schemes followed by the sequence ID matching in proposed work increase the trust level.
- The use of received signal strength indicator (RSSI) in distance estimation effectively predicts whether the trusted node within the communication range or not.
- The log collection-based trust computation, observation schemes and the RSSI estimations in the proposed work increases the packet delivery ratio (PDR), throughput with less delay and false positives compared to the traditional trust-based protocols.

The rest of the paper is systematized as follows: “Related work” section explains the concepts used for trust management in the last few years. “Trust-aware ad-hoc routing protocol” section defines the proposed approach, including neighbor estimation, trust update and distance calculation using RSSI technique. Performance analysis of the proposed approach is explained in “Performance analysis” section. Conclusion and future work are illustrated in “Conclusion and future work” section.

## Related work

This section discusses the influence of trust management protocols on secure data delivery in MANET. The lack of centralized control unit in MANET initiates the cooperation mechanism (nodes rely on the other nodes) to forward the packets. The difficulties in cooperation mechanism made the trust-management as the complex task. Cho et al. (2011) highlighted the factors for trust computation and management. The factors were interactions between the communication networks/information, resource constraints, and dynamics. They combined the notions of social trust and the quality-of-service (QoS) to the compute the trust metric. The direct communication of a specific node with the other nodes within the communication range suffered from the selfish misbehavior. The authenticity, reliability, privacy and trust management were the main issues observed in the MANET architecture. Chaurasia and Tomar (2012) proposed the trust management protocol that considers the certificate of nodes to compute the trust metric and overcome the vulnerabilities. The unique network characteristics and the malicious selfish behavior introduce the challenges in trust computation. Zhu et al. (2014) proposed the probabilistic-based misbehavior detection scheme called iTrust that introduces the periodical trusting authority (TA) to judge the node behavior with the routing evidence and probability checking The design of trust management depends on the prediction of the relationship between the devices (pervasive computing environment) which is the challenging task. Denkoa et al. (2011) reviewed the probabilistic trust management scheme to analyze the interactions and judge the trustworthiness. The maximum trust bias values degrade the application performance adversely. Chen et al. (2014) combined the social trust and quality of service (QoS) constraints to obtain the trust metric for trust bias minimization assurance. They derived optimal protocol by using the combination of the simple lookup table with the interpolation techniques.

The rapid increase in wireless application size maximized the attack densities in MANET. Hence, the suitable plan was required to analyze the sophisticated attack behavior such as insidious and random attacks. The optimized link state routing (OLSR) protocol was suitable in large density MANET with a number of mobile devices interconnection. Tan et al. (2015) proposed trust-based routing mechanism to handle the multi-device problem. They developed trust reasoning model based on fuzzy petri net for maximum trust value path selection. The efficient protocol for trust assurance requires an energy awareness. De Rango et al. (2012) proposed a novel routing strategy to provide the link stability and minimum energy consumption. The mobility of the nodes was not addressed in the link stability routing models. Zhao et al. (2013) proposed a trust management approach in cyclic MANET (cMANET). They considered the time factors in addition to the neighbor relationship to evaluate the performance. The anonymous behavior of nodes leads to security disruption in cyclic models. Gunasekaran and Premalatha (2013) proposed trust-enhanced anonymous on-demand routing protocol to restrict the misuse of anonymity in two methods. The revealing of misbehaving between the users considered in the first method. The multiple claims related to the identification of misbehavior considered in the second method. The large utilization level of social network extended the domain from the internet to mobile raised up pervasive social networking (PSN) which leads to the update in trust management protocols. Yan and Wang (2014) proposed the attribute-based encryption (ABE) model to support the several sequential processes namely data access monitoring of the individual mobile nodes. The filtering of misbehaving nodes in ABE models was achieved by a recommendation based trust management mechanism. The built of trust model based on recommendations was the challenging task due to bad-mouthing and collusion.

Shabut et al. (2015) added the defense mechanism to the traditional recommendation based trust management models. The utilization of clustering technique for filtering of dynamic attacks provided the dishonest recommendations. Artificial intelligence (AI) committee raised up the uncertain reasoning to assure the trust management. Wei et al. (2014) proposed trust management scheme in two aspects namely, direct and indirect observation. The derivation of trust value from Bayesian theory (direct models) and Dempster–Shafer theory (DST) (indirect models). In general, the trust relationship was identified with the help of individual experiences and the recommendations from others. Hence, the number of exchanged messages used for the recommendation were more that leads to excessive energy consumption. Velloso et al. (2010) reduced the message size by a relationship maturity which directly reduced the energy level. Moreover, the extension of maturity concept to the mobile multi-hop networks for minimal energy consumption. The selfish and malicious nodes introduction in wireless sensor network (WSN) made the maturity concept as the difficult one. An establishment of high-security models for ad-hoc networks was long research problem since the vulnerability of nodes and channels were more. Patel et al. (2014) implemented the network intrusion detection systems (NIDS) to analyze these vulnerability issues for assurance of the trust-based routing. But, the reliability and security were less in conventional NIDS system. Jawhar et al. (2014) presented the reliable routing protocol for ad-hoc net to enhance the reliability and security of the MANET environment. But, the trade-off between the high trust level and less mobility was an important requirement in MANET.

Ayday and Fekri (2012) developed the robust trust management protocol based on graph partition algorithm. The data availability and PDR of the graph partition were higher while reducing the latency in the network. The identification of malicious activities was important to assure the optimal security in graph partitioning algorithms. Bu et al. (2011) formulated the malicious activities prediction problem as a multi-objective by using the partial observable Markov decision process (POMDP). They provided the structural results for the combined continuous user authentication and intrusion detection to enhance the security performance in MANET. The practical implementation of MANET dependent on the policies. Due to the presence of malicious nodes in MANET, attacks and unreliability are occurred that limited the trust level. Bijon et al. (2014) adapted the DST which combined the recommendations from multiple devices in the multi-hop environment for a novel trust management scheme implementation. A novel protocol was introduced to reduce the recommendation traffic. The recommendations were prioritized by using a flexible trust computation model. The overall packet flow was reduced to enable the rapid sharing of trust-based information during the presence of contradictory recommendations. The performance validation of trust management protocol was based on trust bias minimization and application performance maximization. Chen et al. (2013) derived a composite metric to evaluate the trust of the mobile nodes in MANET. The trust derived from the social networks was combined with the QoS constraints of the network to obtain the metric. They provided trust management schemes that were not suitable for packet dropping attacks by selfish nodes. The selection of the optimal routing path and the detection of security attacks on that path were difficult in the traditional AODV approaches due to the absence of isolation of malicious nodes. Thanigaivel et al. (2012) proposed trust based routing mechanism called (TRUNCMAN) assured the trust among the nodes and isolate the malicious nodes from normal nodes in order to support the cooperative environment.

Due to the less utilization of third party information in the decentralized control architecture of MANET, the information about the trust is obtained from the peers. Banerjee et al. (2012) proposed reputation-based trust management system for detection and prevention of MANET vulnerabilities. During the fault toleration in MANET, the protocol excluded the malicious and selfish nodes from the network to assure the scalability and robustness. The high-trust assurance is an important requirement in WSN environment. The resource efficiency and the trust assurance were the important factors in the design of WSN. Li et al. (2013) proposed lightweight dependable trust system (LDTS) that employed the clustering algorithms for an effective removal of malicious nodes. Hence, the network consumption level is minimum. The traditional works considered the single dimensional attributes to evaluate the effect of malicious nodes and selfish nodes. The high-reliable path selection is the basic requirement for secure data transfer. Mutlu and Yilmaz (2013) discussed the distributive cooperative trust-based intrusion detection system (DICOTIDS) and evaluated the false positives regarding the effect of malicious and selfish nodes. Bao et al. (2012) presented the dynamic cluster-based hierarchical trust management protocols [geographical routing (GR), flooding based routing (FBR)] that considered the multi-dimensional trust attributes to handle the malicious behavior of network. The problems addressed in the traditional methods were trust assurance through the signal strength estimation was weak, misbehavior report generation, and the presence of trusted node on out of the communication range. This paper discusses the T2AR protocol to handle the problems observed in traditional trust-based protocols.

## Trust-aware ad-hoc routing protocol

**Fig. 1:**
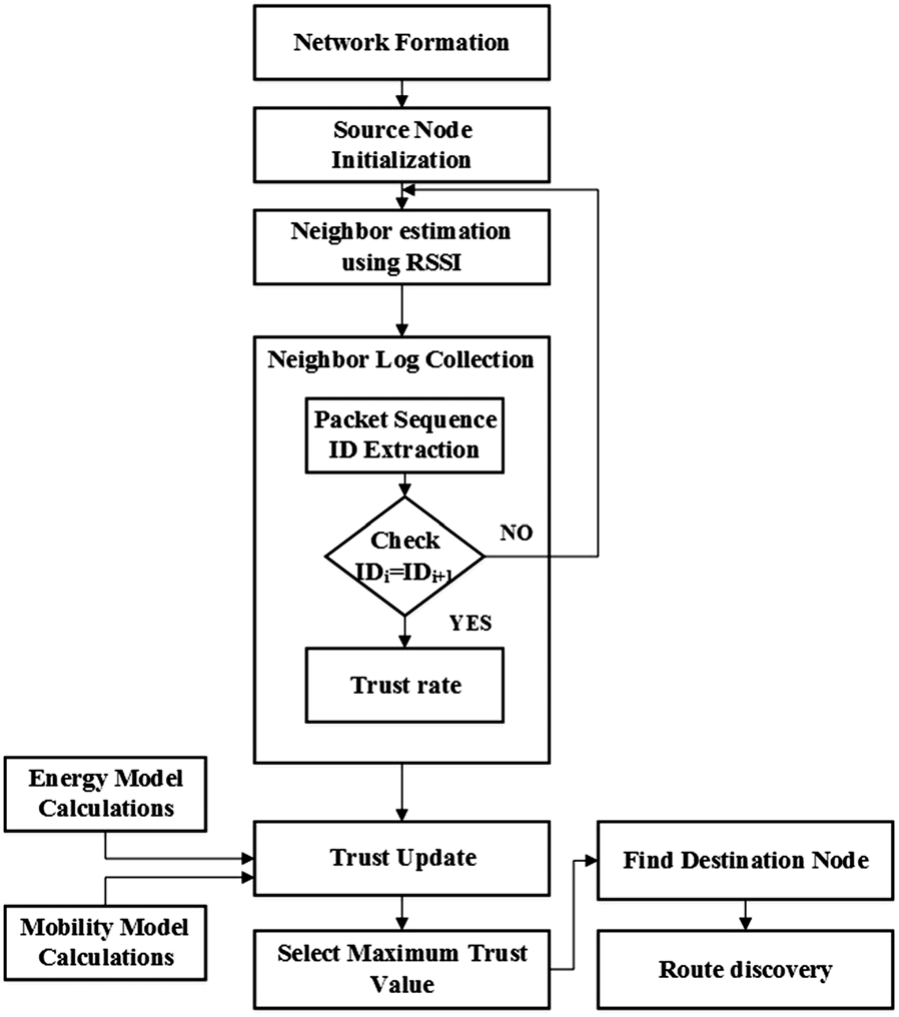
Overall flow diagram of the proposed approach

This section explains about the proposed trust management scheme implementation in detail. The proposed method is mainly used to improve the trust of the nodes in MANET. The following sections describe the processes involved in trust management. The overall flow diagram of the proposed approach is shown in Fig. 1. In our proposed approach, initially a network is formed and the source node is initialized. Then, the proposed algorithm collects the information from the neighbor log reports to know the success/failure rate of packets transfer between the nodes. The trust value is estimated based on the packet sequence ID matching by comparison of log reports of the nodes. Basically, AODV is a reactive routing protocol which establishes the routes whenever required by utilizing the destination sequence numbers to obtain the most recent path. Because of this, AODV determines an up-to-date route to the destination. But, the computed destination nodes are less trustworthy due to the misbehavior report generation. Hence, in proposed work, the trust value is computed through the hybrid estimation of energy, the success rate of packets delivery, and the mobility.

Then, the node with maximum trust value is chosen for packet transfer. With these estimations, the discovered route is reliable, secure and should possess high trust value. Moreover, the distance estimation prior to trust value computation using RSSI assures the selected trust node is within the communication range. The mobile nodes in the MANET have the ability to move in any direction and act as both routers and hosts. Since there is no particular infrastructure, data can be sent from any node to another. The node which sends the packet or data is called as the source node and the node which receives it is called as the destination node. If the trust value of the nodes is found to be high, then transmission of data between them is reliable. In our approach, the trust value is calculated by combining direct and indirect observations. A source node is selected and the neighbor node is extracted by using the RSSI based distance estimation. Then, the trust value of the node is updated based on energy model calculation, packet sequence ID matching rate and mobility estimation. A node having maximum trust value is selected as an intermediate node for packet transfer to the destination node. The proposed scheme is evaluated in terms of PDR, throughput, average delay, the number of false positives. Table 1 represents the definition of variables used in neighbor estimation algorithm.

**Table 1:** Symbols and descriptions

Symbols	Descriptions
d_s,i_	Signal system strength between source node S and current node i
G_i_	ith node in the graph
TR_*i*_	Trust rate
E_s,i_	Energy consumption between source node S and node I
M_i_	Mobility model of ith node
Ei_s,SN_	Overall energy between source node S and node I
Pi_m,n_, Pr_m,n_, Pt_m,n_	Power consumption level during idle, reply and transmission stages
Ti_m,n_, Tr_m,n_, Tt_m,n_	Time required for idle, reply and transmission stages

### Neighbor log collection

Neighborhood estimation is an initial step to compute the trust value of the node. The RSSI-based distance estimation identifies the nodes nearer to the source node. The neighborhood nodes are calculated using the following algorithm:

**Table:** Neighbor log collection

**Input: Node (** ***N*** **), Graph**
**Output: Trust Rate** *TR*_i_
Step 1: Collect the Neighbor Node (NN) list of input node (*N*)
Step 2: Collect the log information of specific NN (*Log*_*N*(*i*)_)
Step 3: Get the packet sequence IDs from the log reports of nodes
Step 4: **For** i = 0 … n **then**//where n = Network Size
Step 5: Calculate d_s,i_ using Eq. (1)
Step 6: $${\mathbf{if}}({\text{d}}_{{{\text{s}},{\text{i}}}} < Range)\,{\mathbf{then}}$$
Step 7: $$PACKET\_ID_{N(i)} = Extract\,ID\,of\,packets (Log_{N(i)} )$$
Step 8: **If** $$PACKET\_ID_{N(i)} = = PACKET\_ID_{N(i + 1)}$$
Step 9: Compute trust rate as *TR*_i_
Step 10: **Else**
Step 11: Goto step 1
Step 12: **End if**
Step 13: **Else**
Step 14: Goto step 1
Step 15: **End if**
Step 16: **End For**

The trust value of the particular node is based on the energy, mobility and trust rate. Hence, the proposed work contains three phases namely trust rate computation, energy model, and mobility model.

#### Trust rate computation

The nodes and the graph are provided as the input values to the neighbor log collection algorithm. Initially, the neighbor node list is constructed for the input node. Then, the information is collected from the log reports of all the nodes participated in the secure data transfer. From the log reports, the packet sequence ID of the particular node is extracted. The distance is estimated by using RSSI method (Saadoune et al. 2014) is as follows:

1 $${\text{d}}_{{{\text{s}},{\text{i}}}} = {\text{RSSI(N,G}}_{\text{i}} )$$

If the computed distance value is less than the communication range, then compare the packet ID of the present node with the ID of next node. If both are equal, then the corresponding trust rate is computed as follows. The trust rate is given by the probability to persist for a certain time span. The trust rate is estimated as follows:

2 $${\text{TR}}_{i} = \frac{1}{3}\left( {{\text{B}}_{{{\text{s}},{\text{i}}}} (H) \times {\text{B}}_{{{\text{i}},{\text{s}}}} ({\text{H}}}) \right) \left( {PS_{R} + RS_{R} + {\text{RQS}}_{R} } \right)$$

3 $${\text{TR}}_{i} = \frac{1}{3}\left( {{\text{B}}_{{{\text{s}},{\text{i}}}} ({\text{H}}) \times {\text{B}}_{{{\text{i}},{\text{s}}}} ({\text{H}})} \right) \left( {\frac{{{\text{NP}}_{s} }}{{{\text{NP}}_{\text{s}} + {\text{NP}}_{\text{f}} }} + \frac{{{\text{NRP}}_{\text{s}} }}{{{\text{NRP}}_{\text{s}} + {\text{NRP}}_{\text{f}} }} + \frac{{{\text{NR}}_{\text{s}} }}{{{\text{NR}}_{\text{s}} + {\text{NR}}_{\text{f}} }}} \right)$$

Belief function $B_{{{\text{S}},{\text{i}}}} ({\text{H}})$ denotes the state of the belief level of the ith node from source node‘s’ and vice versa which varies from 0 to 1. Belief level 0 denotes the unknown status and 1 denotes the known status. Trust rate computation depends on the combination of rate of the successful packet transmission, successful reply rate, and successful request rate.

Packet success rate (*PS*_*R*_) calculated is the ratio between the number of successful packet transmission and overall packet transmission [sum of a number of successful packet transmission (NP_s_) and a number of failed packet transmission (NP_f_)].

Reply success rate (*RS*_*R*_) is the ratio of a number of successful packet transmission and overall reply packet transmission [sum of a number of successful reply packets (NRP_s_) and a number of failed reply packets (NRP_f_)].

Request success rate (RQS_*R*_) is the ratio of successful request transmission to the overall request transmission [sum of a number of successful request (NR_s_) and a number of failed packet transmission (NR_f_)]. The network lifetime improvement depends on the amount of energy preserved and the mobility of the nodes that leads to the construction of energy and mobility models.

#### Energy estimation

Energy is defined as the capacity of the nodes to transfer data. The major functions of the energy model are neighbor sensing and route maintenance. The energy model in our proposed approach is estimated as follows:

4 $${\text{E}}_{{{\text{m}},{\text{n}} }} = \left[ {\left( {{\text{Pi}}_{{{\text{m}},{\text{n}}}} \times {\text{Ti}}_{{{\text{m}},{\text{n}}}} } \right) + \left( {\hbox{Pr}_{{{\text{m}},{\text{n}}}} \times {\text{Tr}}_{{{\text{m}},{\text{n}}}} } \right) + \left( {{\text{Pt}}_{{{\text{m}},{\text{n}}}} \times {\text{Tt}}_{{{\text{m}},{\text{n}}}} } \right)} \right]$$

After selection of trusted node for packet transmission, selected node requires updating their energy level for further packet transmission. Overall energy (Ei_m,n_) will be updated by using following equation

5 $${\text{Ei}}_{{{\text{m}},{\text{n}}}} = {\text{Ei}}_{{{\text{m}},{\text{n}}}} - {\text{E}}_{{{\text{m}},{\text{n}}}}$$

#### Mobility function

Mobility function describes the movement of the mobile nodes and it contains the moving speed of a node, direction. The distance between the nodes is calculated using a constant value ‘k’ and power required for transmission/reception as follows.

6 $$d = \sqrt[4]{{{\text{k}} \cdot {\text{Pt}}/\Pr }}$$

Neighbor velocity is calculated by,

7 $$\bar{V} = \Delta {\text{d}}/\Delta {\text{t}}$$

Depends on the velocity parameter, the neighbor node position level with respect to selected node is obtained using,

$${\text{Direction}} = \left\{{\begin{array}{*{20}l} {{\bar{\text{v}}} > 0} \hfill &\quad {outward} \hfill \\ {{\bar{\text{v}}} = 0} \hfill &\quad {\text{static}} \hfill \\ {{\bar{\text{v}}} < 0} \hfill &\quad {inward} \hfill \\ \end{array}} \right.$$

The mobility function is obtained from

8 $$M_{i} = \bar{V}TR_{i} + d$$

By using the estimated values of energy, trust rate, and the mobility from (3), (4) and (8), the trust value of the node is computed as follows:

9 $${\text{TC}}_{{{\text{s}},{\text{i}} }} = {\text{E}}_{{{\text{s}},{\text{i}}}} + TR_{\text{i}} - {\text{M}}_{\text{i}}$$

From the computed trust value, the node with maximum trust value is chosen for data or packet transfer. Once the destination is reached, route discovery has to be done. Otherwise, again the neighbor estimation has to be done. This method examines the route between the source and the neighbor nodes in the network. If the distance between the source and neighbor node lies within the coverage area, then it is added to the NN list and collects the log reports for the computed neighbor node. The packet sequence ID from the log reports is extracted and compared with other nodes. If both the IDs are matched, then the trust rate is computed by using the request, reply, packet delivery rate and the computed trust rate is given as the input parameter to the trust update process.

### Trust update

There may be malicious or misbehaving nodes in the MANET and so, trust between nodes is significant for packet transfer. The presence of malicious nodes causes the packet dropping and incorrect packet forwarding to the nodes in general. In this paper, the effect of malicious nodes under packet dropping is considered. The trust assurance via direct and indirect observational schemes is efficiently reduces the packet drops in proposed work.

#### Direct observation

In this scheme, an observer node can directly estimate the trust value by using the Bayesian framework with the assumption of an observer node overhear the forwarded packets and compared with the original packets to find the malicious behavior. The distribution function with shape parameters *α* > 0, *β* > 0 and the random variable 0 < *θ* < 1 follows the beta function as follows:

10 $$Beta(\theta ;\alpha ,\beta ) = \frac{{\theta^{\alpha - 1} \left( {1 - \theta } \right)^{\beta - 1} }}{{\mathop \smallint \nolimits_{0}^{1} \theta^{\alpha - 1} \left( {1 - \theta } \right)^{\beta - 1} d\theta }}$$

The degree of belief functions between the nodes *B*_*s*,*i*_ is defined by the expectation function or punishment factor as follows:

11 $$B_{s,i} = E_{n} (\varTheta ) = \frac{{\alpha_{n} }}{{\alpha_{n} + \beta_{n} }}$$

The more weight on punishment factor specifies the huge misbehavior is observed which leads to less trust value. By using this type of factor measurement, the isolation of malicious node from the normal node is achieved. The punishment factor-based deduction refers the trust rate value as

12 $$TR = E_{n} (\varTheta )$$

The observation scheme based on malicious behavior identification leads to less trust in traditional protocols. But, the trust-aware routing protocols implementation in this paper computes the trust rate via sequence ID matching assures the secure data transfer between the nodes.

#### Indirect observation

The Dempster’s Shafter theory (DST) based indirect observation computes the belief functions for three sets as follows:

$$H = \{ trust\} , \quad \bar{H} = \{ untrust\} ,\quad U = \{ trust\,or\,untrust\}$$

The belief function for the above sets if the node A observed node B as trusted node is as follows:

13 $$\begin{aligned} & B(H) = TR \\ & B\left( {\bar{H}} \right) = 0 \\ & B(U) = 1 - TR \\ \end{aligned}$$

The belief function for the above sets if the node A observed node B as untrusted node is as follows:

14 $$\begin{aligned} & B(H) = 0 \\ & B\left( {\bar{H}} \right) = 1 \\ & B(U) = 1 - TR \\ \end{aligned}$$

**Table:** Trust update

**Input**: Source node N_s_, Destination node N_d_, Graph G
**Output**: Routing Path R_p_
**Procedure:**
$$TR_{\text{i}} \leftarrow {\text{NeighborLog}}\,{\text{Collection}}({\text{N}}_{\text{s}} , {\text{G}})$$
**For** i = 0, 1, 2 … n **Then**
Calculate trust value by using Eq. (9)
**End For**
SN = MAX (TC_s_)
$${\text{R}}_{\text{p}} \leftarrow {\text{R}}_{\text{p}} \cup {\text{SN}}$$
$${\text{G}} \leftarrow {\text{G}}^{\prime } \notin {\text{SN }}$$
**If** $$({\text{N}}_{\text{d}} ! = {\text{SN}})$$ **then**
Update Energy Ei_s,SN_
N_s_ = SN
Repeat from *TR*_i_
**End If**
**Return** R_p_

During the update process, the trust rate (*TR*_i_) is computed from the neighbor log collection process initially. Then, for each node, the trust value is computed by using the energy model (E_s,i_), Trust rate (*TR*_*i*_) and mobility model (M_i_) by using the Eq. (9). The node with high trust value (SN) is selected and added to the routing path. Then, the graph is updated with the remaining nodes. Then, it checks whether the selected node is the destination or not. If the destination node is not equal to the selected node having maximum trust value, then the selected node is considered as the source node. Then, the energy of the node is updated and repeat the neighbor log collection process. If the destination node is found to be equal to the node with maximum trust value, then the routing path is determined.

## Performance analysis

In this section, the performance of the proposed T2AR approach and the existing trust-based routing mechanism on non-cooperative environment of MANET (TRUNCMAN) (Thanigaivel et al. 2012), reputation-based trust management protocol (RBT) (Banerjee et al. 2012) trust based GR approaches (Bao et al. 2012) and distributed cooperative trust-based intrusion detection system (DICOTIDS) (Mutlu and Yilmaz 2013) compared. The metrics used for the performance evaluation of the proposed T2AR approach and existing approaches are PDR, throughput, average delay and false positives. The proposed system is simulated with the network simulator-2 (NS-2) with the simulation parameters of Table 2.

**Table 2:** Simulation parameters

Simulation parameters	Values
Application protocol	CBR
CBR transmission time (s)	1–100
CBR transmission interval (s)	0.5
Packet size (bytes)	512
Transport protocol	UDP
Network protocol	IPv4
Routing protocol	T2AR
MAC protocol	IEEE 802.11
Physical protocol	IEEE 802.11b
Data rate (Mbps)	2
Transmission power (dBm)	0.14
Radio range (m)	180
Propagation path loss model	Two ray
Simulation area	500 × 500
Number of nodes	100
Simulation time (s)	600

### PDR

PDR is the ratio of the number of data packets received by a destination node and the number of data packets generated by a source node.

15 $${\text{PDR}} = \frac{{{\text{Number}}\,{\text{of}}\,{\text{packets}}\,{\text{received}}}}{{{\text{Number}}\,{\text{of}}\,{\text{packets}}\,{\text{transmitted}}}} \times 100$$

**Fig. 2:**
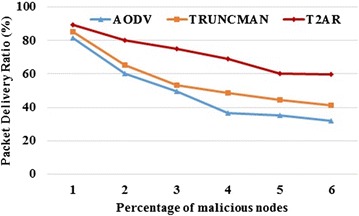
PDR versus the percentage of malicious node

**Fig. 3:**
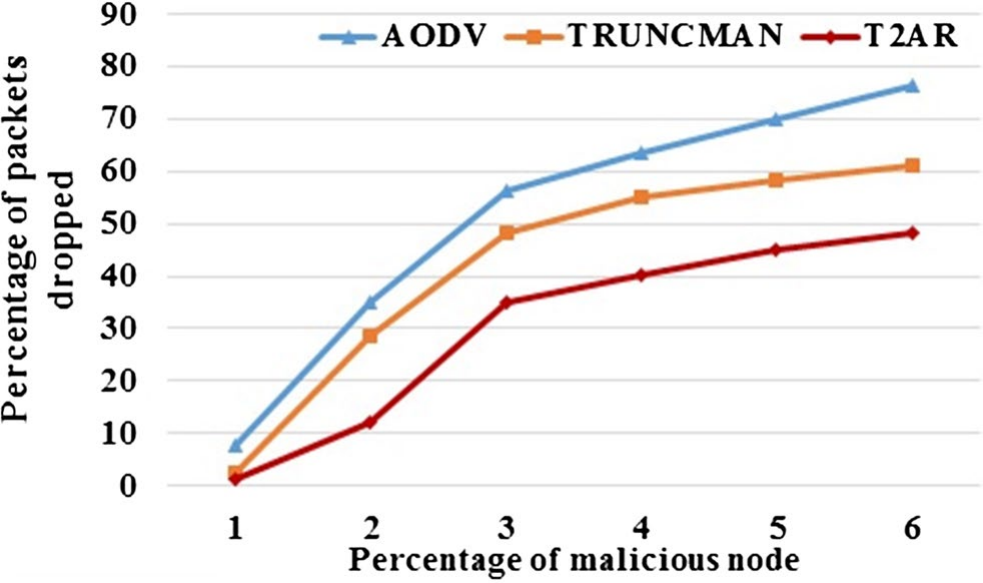
Packets dropped versus percentage of malicious nodes

**Fig. 4:**
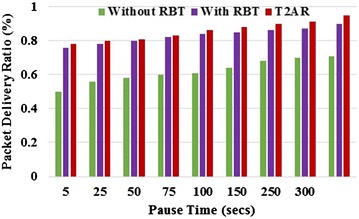
Packet delivery ratio versus pause time

Since the buffer is free, the packets are delivered quickly within a small amount of time interval. The trust-based routing calculation easily detects the misbehavior of malicious nodes, the PDR of the proposed approach is higher than the existing approaches. Figures 2 and 3 shows the proposed scheme obtains better PDR compared with existing TRUNCMAN and Fig. 4 shows the variation of throughput in RBT approaches on an increase in the percentage of malicious nodes and the simulation time respectively. On comparing the proposed T2AR with the TRUNCMAN and AODV over the percentage of malicious nodes, the proposed T2AR provides 8.93 and 4.64 % better than the AODV and TRUNCMAN for low malicious ratio values and it provides 45.97 and 30.68 % better for high malicious ratio respectively. Similarly, the comparative analysis of proposed T2AR with the TRUNCMAN and AODV on packets dropped conveys that the T2AR provides 86.11 and 54.58 % less for low malicious ratio and it offers 36.9 and 21.4 % less for high malicious ratio respectively due to the tri-series (energy, mobility, trust rate) trust modeling.

From the Fig. 4, it is observed that the proposed T2AR provides the 35.9 and 2.56 % better for low simulation period values compared to without RBT and with RBT. Similarly, the T2AR provides 25.26 and 5.26 % better performance for high simulation period. The comparative analysis between the proposed T2AR and the existing approaches depicts the locational information update-based trust rate computation assures the high-security data transfer and improves the PDR.

### Throughput

**Fig. 5:**
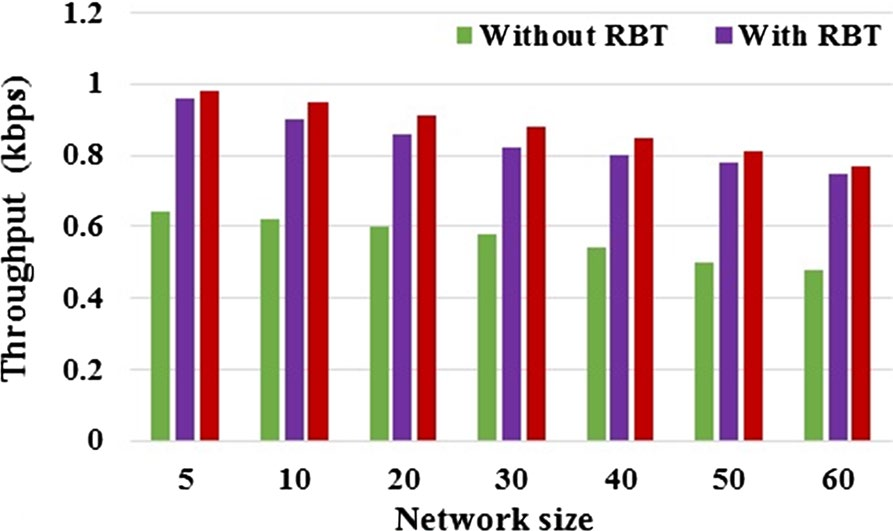
Throughput versus network size

Throughput is defined as the total size of data packets correctly received by a destination node in every second. It gives the information whether the data packets are correctly delivered to the destinations are not. By estimating the trust value of the nodes, the occurrence of malicious attacks is prevented. Figure 5 shows that the throughput of T2AR increases compared with RBT and without RBT for variations in network size.

From the figure, the T2AR approach yields 34.69 and 2.04 % better throughput for low network size values and it offers 37.66 and 2.6 % better for large network size.

### Average end-to-end delay

The average end-to-end delay is defined as the mean value of end-to-end delay between the source and destination nodes. End-to-end delay is the total amount of time taken for transmitting a packet from the source to the destination. It includes the delay caused by route discovery process and the queue in the data packet transmission.

**Fig. 6:**
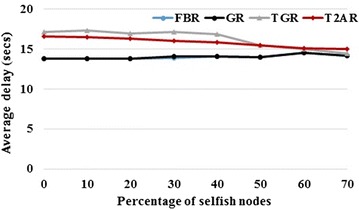
Average end-to-end delay versus percentage of selfish nodes

This is calculated by subtracting the time at which the data packet was transmitted by the source node from the time at which the data packet arrived at the destination node. Figure 6 shows that the proposed scheme obtains lower average end-to-end delay than the existing trust based GR scheme, FBR and GR without trust over the various percentage of selfish nodes.

The existing FBR/GR models offer the least delay performance compared to TGR model. But, the inclusion of trust observation based on neighbor log collection optimizes the delay performance between FBR and TGR. The comparative analysis depicts that the proposed T2AR provides 16.67 and 5.27 % better than FBR models for low and high-percentage of selfish nodes respectively.

### False positives

**Fig. 7:**
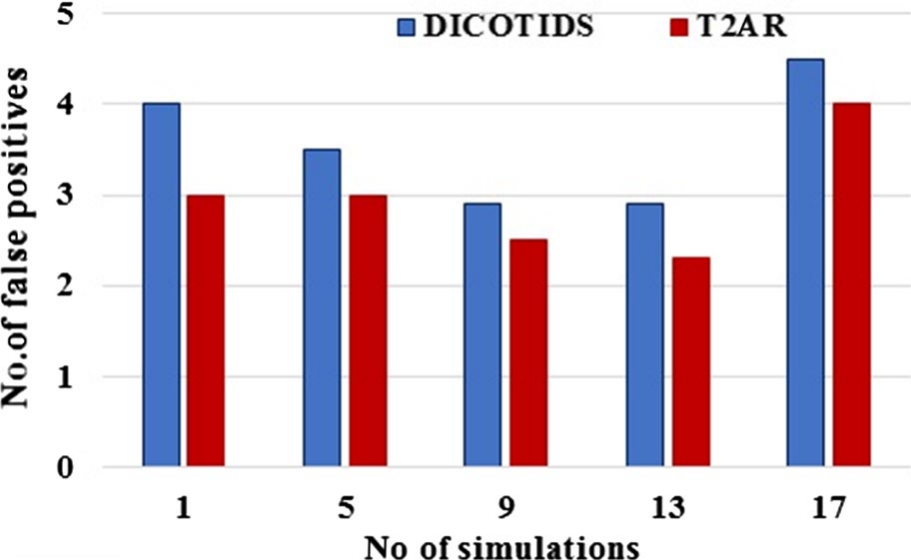
False positives versus number of simulations

The detection probability of misbehaving nodes against the total number of nodes constitute false positives. Figure 7 shows the comparison result of the false positive with the simulations. The false positive seems to reduce efficiently, with respect to the increase in the number of simulations.

The graphical illustration in Fig. 7 depicts that the proposed T2AR scheme reduces the false positive performance by 33.33 and 12.5 % for low and high simulation count values respectively.

## Conclusion and future work

This paper proposed a T2AR protocol for the trust level improvement between the nodes in MANET and enhanced the secure data transfer performance. The proposed method modified the traditional AODV routing protocol with the constraints of stability of connection establishment and energy, mobility based malicious behavior prediction. The trust value is calculated on the basis of energy, mobility and RSSI-based distance measurement. The information about the trust assurance is obtained from peers in reputation based routing protocols provided less PDR and throughput with an increase of malicious nodes ratio. To improve this, the T2AR is proposed which gathered the log information from the neighbor nodes through the direct and indirect observation schemes. The ID matching based trust rate calculation improves the trust level compared to conventional models. The overall performance of the proposed approach is compared with the existing trust-based routing management in a non-cooperative environment in MANET (TRUNCMAN), reputation-based trust-aware routing protocols (RBT), trust-based GR, FBR and DICOTIDS. The proposed approach achieved high throughput and PDR and optimal end-to-end delay and less false positives. Our future work will include the security enhancement using location key management protocol. Enhancing the security by using location and key based security scheme is considered as the future work.
